# Ultradeformable liposomal delivery of vismodegib modulates its biological activity in melanoma cells

**DOI:** 10.3389/fmedt.2026.1826970

**Published:** 2026-06-05

**Authors:** Maria Natalia Calienni, Cristian Sandoval-Acuña, Petra Potomová, David Emanuel Ybarra, Jaroslav Truksa, Jorge Montanari

**Affiliations:** 1 Universidad Nacional de Hurlingham (UNAHUR), Laboratorio de Nanosistemas de Aplicación Biotecnológica (LANSAB), Villa Tesei, Buenos Aires, Argentina; 2 Consejo Nacional de Investigaciones Científicas y Técnicas (CONICET), Buenos Aires, Argentina; 3 Comisión de Investigaciones Científicas de la Provincia de Buenos Aires (CIC), La Plata, Argentina; 4 Departamento de Ciencias Básicas, Facultad de Medicina, Universidad de La Frontera, Temuco, Chile; 5 Laboratory of Tumour Resistance, Institute of Biotechnology, Czech Academy of Sciences, Vestec, Czechia

**Keywords:** vismodegib, hedgehog signaling, drug repurposing, melanoma, ultradeformable liposomes, dendrimers, nano-based drug delivery

## Abstract

**Introduction:**

Vismodegib (VDG), a Smoothened inhibitor approved for basal cell carcinoma, has potential for repurposing in other tumors in which Hedgehog (Hh) signaling contributes to malignancy. However, systemic VDG treatment is associated with relevant adverse effects, and its effective delivery to cutaneous targets remains challenging, supporting the exploration of drug delivery systems for its administration.

**Methods:**

Here, we first screened the response to VDG across four murine tumor cell lines-B16 melanoma, 4T1 mammary carcinoma, Colon-26 colon carcinoma, and LLC1 Lewis lung carcinoma-and then evaluated whether nanocarrier-based delivery could modify its activity in melanoma cells.

**Results:**

Free VDG produced moderate effects across the screened models, with B16 emerging as the most responsive model showing evidence of growth inhibition together with modulation of Hh-related markers. In this setting, encapsulation of VDG into ultradeformable liposomes (UDL), specially designed for topical application, significantly changed the biological response to treatment, producing a stronger cytotoxic response, concentration-dependent inhibition of cell population expansion, and increased accumulation of dead cells compared with the free drug. An exploratory PAMAM G4.5 dendrimer-based formulation did not show detectable biological effects at the tested concentration, which was constrained by the need to maintain subtoxic dendrimer levels. Although no significant transcriptional changes were detected at the analyzed time point, reduced Gli-1 expression at higher concentrations suggested some degree of Hh pathway modulation by UDL-VDG. However, the enhanced biological effect of UDL-VDG could not be explained solely by canonical Hh inhibition, and responses varied across melanoma models.

**Discussion:**

Overall, these results show that ultradeformable liposomal delivery potentiates the in vitro anti-melanoma activity of VDG and supports its further exploration as a repurposing strategy for candidate non-metastatic cutaneous melanoma. More broadly, these findings support the rationale for topical nanocarrier-based delivery as an approach that could potentially improve local drug availability while helping to reduce some of the limitations associated with systemic administration.

## Graphical Abstract

**Figure:**
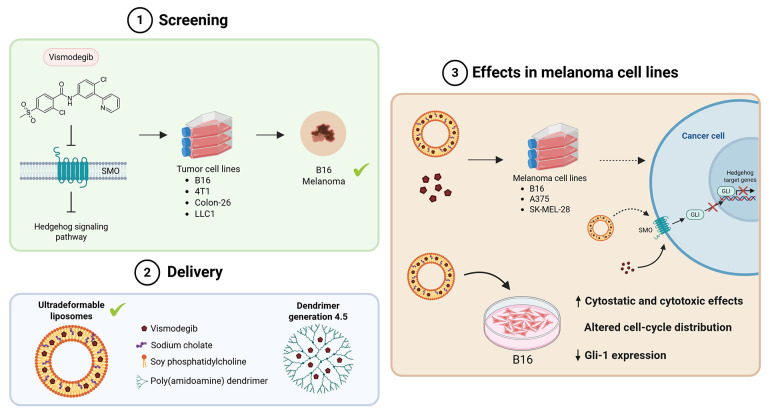
Schematic overview of the experimental design and main findings of the study. Created in BioRender. Calienni, M. N. (2026) https://BioRender.com/y2qb1b1.

## 1 Introduction

Nearly twenty million people are diagnosed with cancer each year worldwide, with more than 1.5 million corresponding to skin cancer. Among these, melanoma represents the most aggressive form, although it is less frequent than non-melanoma skin cancers (1). Despite recent advances in immunotherapy and targeted therapies, a significant proportion of patients experience tumor progression or relapse, highlighting the need to develop new therapeutic strategies or optimize the use of existing drugs (2–4). In this context, drug repurposing and the development of advanced delivery systems have emerged as promising approaches to improve the efficacy of already approved compounds and broaden their applicability across various cancer types, including skin tumors (5–10).

Among the signaling pathways involved in tumorigenesis and progression of multiple neoplasms is the Hedgehog (Hh) signaling pathway. Particularly, this pathway is aberrantly reactivated in both melanoma and non-melanoma skin cancer, contributing not only to tumor growth but also to therapy resistance (10–15). A little over a decade ago, Vismodegib (VDG) was approved by global regulatory agencies as the first Hh inhibitor, a selective Smoothened receptor (SMO) antagonist that blocks downstream Hh signaling and reduces the activity of Gli family transcription factors (16). Since then, it has been approved as a treatment for basal cell carcinoma and is administered orally at 150 mg per day (17–19). However, its application in other solid tumors has shown variable results, partly due to differences in tumor dependence on the Hh pathway and the activation of compensatory mechanisms that can limit therapeutic efficacy. These observations have spurred research into the potential repurposing of VDG in other tumor models and the search for strategies to improve its biological activity (20–22). In this regard, its systemic distribution leads to adverse effects that compromise quality of life and treatment adherence, while only a minimal fraction of the drug effectively reaches skin tumors (23).

Localized administration to cutaneous tumors could benefit from the use of delivery systems capable of improving topical penetration and promoting sustained drug exposure within the tumor microenvironment. In this regard, nanostructured drug delivery systems have emerged as promising tools for optimizing the delivery of hydrophobic compounds, such as VDG (24, 25). By the end of the past decade, several strategies aimed at local VDG delivery began to appear in the literature, including the incorporation of the molecule into nanomaterials to constitute topical drug-delivery systems, designed to improve cutaneous penetration and reduce systemic exposure (26). Liposomes, ethosomes, transfersomes, dendrimers, and related nanosystems have demonstrated the capacity to enhance the apparent solubility of VDG, facilitate its tissue permeation, and support targeted or localized therapeutic strategies (27–29). These advances place such platforms at the forefront of efforts to refine and repurpose VDG, while also contributing to the broader landscape of next-generation MedTech developments. In particular, the reactivation of the Hh pathway in several other types of skin cancer, including melanoma, makes VDG an especially interesting candidate for drug repurposing, prompting further investigation into the sensitivity of different tumor types to this drug. Furthermore, the complementary action of VDG in repurposing strategies involving combination therapies with other drugs has also been highlighted (30).

Despite these advantages, significant gaps remain-stemming from the tunable architecture of soft nanomaterials, their capacity to efficiently load hydrophobic molecules such as VDG, and their influence on cellular uptake. In particular, there is still limited understanding of how distinct drug-nanocarrier systems differentially modulate biological interactions in normal and tumor cells, as well as drug-mediated signaling pathways. Ultradeformable liposomes (UDL), characterized by high elasticity at physiological temperature that enables penetration of the *stratum corneum* of the skin, and poly(amidoamine) (PAMAM) dendrimers, noted for their small size and high solubilization capacity, have been extensively studied as carriers for VDG (31, 32), since they can also function as topical delivery systems (33, 34). However, studies assessing how these nanocarriers modulate cellular responses and the mechanistic effects of the drug remain scarce. Furthermore, given that the Hh pathway activates genes related to pluripotency and chemoresistance (35), it is crucial to determine whether the delivery of the drug through nanosystems influences the modulation of the stemness profile, which can be a key determinant of resilience in melanoma (36, 37).

Considering this background, the comparative evaluation of different delivery systems for VDG could provide relevant insights to optimize its therapeutic performance and explore new applications in cutaneous tumors. In addition, analyzing its biological activity across different tumor models may help identify cellular contexts in which inhibition of the Hh signaling pathway exerts the greatest antiproliferative impact. In this study, we evaluated the biological activity of VDG in several murine tumor cell lines associated with Hh signaling and investigated whether its encapsulation in nanocarriers could enhance its antiproliferative effects. For this purpose, the cellular response to the free drug was compared with formulations based on UDL and generation 4.5 PAMAM dendrimers (DG4.5) with terminal carboxylates, with a particular focus on melanoma models. Cytotoxicity and antiproliferative assays, as well as pathway-level analyses, were performed to determine how nanocarrier incorporation influences the biological activity of VDG across different tumor cells.

## 2 Materials and methods

### 2.1 Materials

Vismodegib (2-chloro-N-[4-chloro-3-(pyridin-2-yl)phenyl]-4-(methylsulfonyl)benzamide) and a C18 HPLC column were generously provided by Roche S.A.Q. e I. (Buenos Aires, Argentina). Phospholipon® 90 G (≥94.0% phosphatidylcholine) was purchased from Lipoid GmbH (Ludwigshafen/Rhine, Germany). PAMAM dendrimers generation 4.5 in methanolic solution, molecular biology grade water, DMEM medium, Penicillin-Streptomycin solution, sodium cholate, sodium deoxycholate, Tris-HCl, sodium chloride, sodium dodecyl sulfate (SDS), ethylenediaminetetraacetic acid (EDTA), Tween-20, NP-40, and other analytical-grade reagents were supplied by Sigma-Aldrich (St. Louis, MO, USA). Gibco™ fetal bovine serum (FBS), AlamarBlue™ HS Cell Viability, SYTOX™ Green Nucleic Acid Stain, Vybrant™ DyeCycle™ Violet Ready Flow™, Pierce™ BCA Protein Assay kit, and RevertAid First Strand cDNA Synthesis kit were from Thermo Fisher Scientific (Waltham, MA, USA). Radiance Plus kit for chemiluminescent Western blotting was obtained from Azure Biosystems, Inc. (Dublin, CA, USA). Albumin fraction V was from Carl Roth (Karlsruhe, Germany). Protease inhibitors mix M, phosphatase inhibitor mix, glycogen, and milk powder were purchased from SERVA Electrophoresis (Heidelberg, Germany). RNAzol and 4-bromoanisol were from Molecular Research Center (Cincinnati, OH, USA). L-glutamine was obtained from PAN-Biotech (Aidenbach, Germany). HOT FIREPol® EvaGreen® qPCR Mix Plus was from Solis BioDyne (Tartu, Estonia). Acetonitrile of HPLC grade and chloroform were purchased from J. T. Baker® (Buenos Aires, Argentina). Methanol and dimethyl sulfoxide (DMSO) were obtained from Biopack (Buenos Aires, Argentina).

### 2.2 Methods

#### 2.2.1 Preparation and characterization of vismodegib-loaded UDL

UDL containing VDG (UDL-VDG) were prepared following a previously reported procedure (31). Briefly, soybean phosphatidylcholine, sodium cholate, and VDG were combined at a mass ratio of 40:7:1.4, dissolved in a chloroform–methanol mixture (1:1, v/v), and evaporated under reduced pressure to form a thin lipid film. The dried film was flushed with nitrogen and rehydrated in Tris–HCl buffer (10 mM, pH 7.4, containing 0.9% w/v NaCl) to obtain a liposomal suspension, which was subsequently extruded ten times through polycarbonate membranes (100 nm pore size) under nitrogen pressure to yield unilamellar vesicles (LIPEX® Flow 10 mL Thermobarrel Extruder, Evonik, Essen, Germany).

UDL-VDG were characterized to confirm that their physicochemical properties were consistent with those previously reported. Mean particle size, polydispersity index, and zeta potential were determined using a Zetasizer Pro Blue (Malvern Panalytical Ltd., UK). The concentration of encapsulated VDG was quantified after vesicle disruption in acetonitrile, followed by HPLC analysis, as previously described (31).

#### 2.2.2 Preparation of DG4.5-vismodegib complexes

VDG was also complexed to DG4.5 (DG4.5:VDG) in order to explore an alternative nanocarrier strategy. The complexation procedure and physicochemical characterization are described in detail in the Supplementary Material.

#### 2.2.3 Cell culture

The murine cell lines 4T1 (ATCC CRL-2539), B16 (ATCC CRL-6475), Colon-26 (ATCC CRL-2638), and LLC1 (ATCC CRL-1642), and the human melanoma cell lines SK-MEL-28 (ATCC HTB-72) and A375 (ATCC CRL-1619) were obtained from ATCC (Manassas, VA, USA). Cells were maintained in a humidified incubator at 37 °C with 5% CO_2_ in DMEM supplemented with 10% FBS, 100 U/mL penicillin, 100 µg/mL streptomycin, and 2 mM L-glutamine. Cultures were routinely screened for mycoplasma contamination using the MycoAlert® PLUS Detection Kit (Lonza, Switzerland) and were used only within three months after thawing.

The murine cancer cell lines were used as the primary experimental models throughout the study. Human melanoma cell lines were included in selected assays using free and liposome-encapsulated VDG.

#### 2.2.4 Cytotoxicity determination

Cells were seeded in 96-well plates at a density of 1 × 10^4^ cells per well and incubated overnight to allow attachment. The following day, the test solutions were added. Since free VDG is poorly soluble in culture medium, it was dissolved in DMSO, and the maximum DMSO concentration tested was 1%; therefore, a vehicle control was included. After 24 or 48 h of incubation, cell viability was assessed using the Alamar Blue HS assay. Fluorescence was recorded at 560 nm excitation and 590 nm emission using a microplate reader (Infinite M200, Tecan, Switzerland). Dose-response curves were fitted using a four-parameter logistic model with variable slope, and IC_50_ values were calculated using GraphPad Prism® 8.0.1 software (GraphPad Software, San Diego, CA, USA).

#### 2.2.5 Apoptosis and autophagy modulation

To investigate whether the treatments modulated apoptosis- and autophagy-related pathways, protein levels were determined by SDS-PAGE and Western blot. Cells were seeded in 100 mm Petri dishes at a density of 1 × 105 cells/mL and allowed to adhere overnight. The following day, selected concentrations of free VDG or UDL-VDG were applied, and cells were incubated for 24 h. Cells were washed with phosphate-buffered saline (PBS) and lysed in conventional RIPA buffer (150 mM NaCl, 50 mM Tris pH 8, 1% NP-40, 0.1% SDS, 1 mM EDTA, 0.5% sodium deoxycholate) containing protease and phosphatase inhibitor cocktails. Protein concentrations were determined using the BCA assay, and equal amounts of protein (30 µg) were resolved by SDS-PAGE and transferred to 0.2 μm PVDF membranes (Thermo Fisher Scientific). Membranes were rinsed with Tris-buffered saline containing 0.05% Tween-20 (TBS-T) and blocked with 5% non-fat milk in TBS-T for 1 h. After washing, membranes were incubated overnight with primary antibodies against LC3B, Beclin-1, p62, PARP, cleaved PARP, or β-actin conjugated to HRP (Supplementary Table S1). Primary antibodies were diluted 1:1000, except for HRP-conjugated β-actin (1:5000), in 5% albumin fraction V prepared in TBS-T. After incubation, membranes were washed three times with TBS-T and incubated for 1 h with horseradish peroxidase (HRP)-conjugated secondary antibodies against mouse or rabbit IgG (H + L) at a 1:5000 dilution in 1% milk in TBS-T. Protein bands were detected by enhanced chemiluminescence using an HRP-based substrate and visualized with an Azure c600 imaging system (Azure Biosystems). Bands were quantified by densitometry using ImageJ software (NIH, USA; v1.54p), normalized to β-actin, and expressed as fold change relative to untreated controls. In addition, the LC3-II/LC3-I ratio was calculated.

#### 2.2.6 Cell cycle assessment

Cell cycle distribution was assessed by flow cytometry as previously described (38). Cells were seeded in 6-well plates at a density of 1 × 10^5^ cells per well and allowed to adhere overnight. The following day, selected concentrations of the treatments were added. After 24 h of incubation, cells were washed with PBS and harvested by trypsinization. Detached cells were collected in fresh culture medium and centrifuged at 350 × *g* for 5 min at 4 °C. Pellets were resuspended in medium containing Vybrant DyeCycle Violet Stain and incubated for 30 min at 37 °C. Fluorescence was analyzed using a BD LSRFortessa™ flow cytometer (BD Biosciences, Franklin Lakes, NJ, USA) with excitation at 405 nm and emission at 437 nm. A total of 20,000 events per sample were acquired, and data were processed using FlowJo software (BD Biosciences).

#### 2.2.7 Effect on hedgehog signaling and stemness-related markers

The effects of free VDG and UDL-VDG on Hh signaling and stemness-related markers were investigated by Western blot analysis and RT-qPCR.

For Western blot analysis, the same procedure described in Section 2.2.5 (Apoptosis and autophagy modulation) was followed. Membranes were incubated overnight with primary antibodies against SHH, SMO, Gli-1, CD44, or β-actin (Supplementary Table S1).

For RT-qPCR studies, cells were seeded in 60 mm Petri dishes at a density of 5 × 10^4^ cells/mL and allowed to adhere overnight. The following day, selected drug concentrations were applied, and cells were incubated for an additional 24 h. Total RNA extraction was performed using RNAzol according to the manufacturer's instructions. RNA purity and concentration were assessed using a Tecan microplate reader. A total of 1 μg of RNA was reverse-transcribed into cDNA using the RevertAid First Strand cDNA Synthesis Kit (Thermo Fisher Scientific) with random hexamer primers, following the manufacturer's protocol. Quantitative PCR was performed using HOT FIREPol EvaGreen qPCR Mix in 384-well plates on a CFX384 Thermal Cycler (Bio-Rad). Primer sequences are listed in Supplementary Table S2. Thermal cycling conditions consisted of an initial denaturation at 95 °C for 12 min, followed by 40 cycles of denaturation at 95 °C for 10 s, annealing at 60 °C for 25 s, and extension at 72 °C for 20 s. Target gene expression was normalized to the housekeeping gene 12S rRNA, and relative expression levels were calculated using the ΔΔCt method using GenEx software version 6 (MultiD Analyses AB, Gothenburg, Sweden).

#### 2.2.8 Proliferation and cell death kinetics

Cells were seeded in 96-well plates at a density of 2 × 10^3^ cells per well and allowed to adhere overnight. The following day, selected concentrations of the test compounds were added and maintained throughout the entire incubation period. Cell proliferation and death were monitored by real-time imaging using an Etaluma LS720 microscope (Etaluma, Carlsbad, CA, USA) or an IncuCyte® S3 system (Sartorius, Göttingen, Germany), as previously described (39). Images of each well were automatically acquired every 3 h for up to 72 h. Cell proliferation and death were quantified using Lumaview or IncuCyte software, which provided measurements of confluence and the number of non-viable cells over time. Cell proliferation was assessed by measuring phase-area confluence. To assess cell death, the fluorescent dye SYTOX^™^ Green was used at a final concentration of 150 nM, with excitation and emission wavelengths set at 483 and 503 nm, respectively. Cell death was quantified by object counting. For data normalization, dead cells present immediately after compound addition were subtracted, establishing a baseline of zero at time zero. Additionally, dead cell counts at each time point were normalized to the corresponding confluence area.

#### 2.2.9 Statistical analysis

Unless otherwise indicated, experiments were performed with three independent biological replicates and data are presented as mean ± SD. Statistical comparisons between experimental groups and controls were performed using two-way ANOVA followed by Dunnett's *post hoc* test in GraphPad Prism 8.0.1 (GraphPad Software, Boston, USA). Differences were considered statistically significant at *p* < 0.05.

## 3 Results and discussion

### 3.1 Screening of VDG activity across murine cancer cell lines

To explore the anti-cancer potential of VDG beyond basal cell carcinoma, we initially evaluated its activity across a panel of murine cancer cell lines derived from tumor types in which Hh signaling has been reported to contribute to disease progression or therapy resistance. The selected cell lines included 4T1 mammary carcinoma, B16 melanoma, Colon-26 colon carcinoma, and LLC1 Lewis lung carcinoma, representing distinct tissue origins and biological behaviors. This *in vitro* screening enabled a comparative assessment of VDG effects on cell viability, cell-cycle distribution, and selected molecular markers.

**Figure 1:**
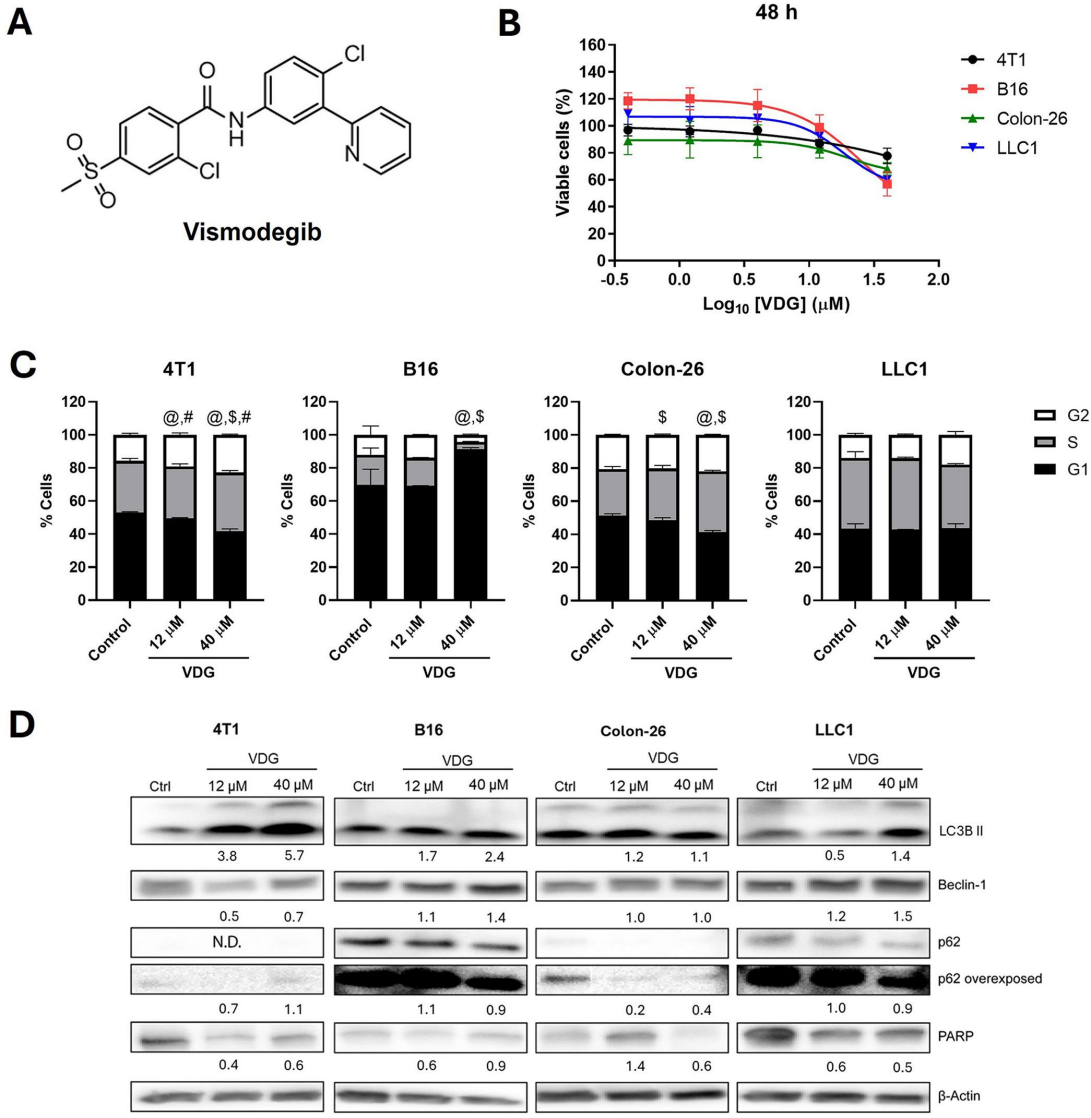
Effects of VDG on cell viability, cell cycle progression, and autophagy- and apoptosis-related proteins in 4T1, B16, colon-26, and LLC1 murine cancer cell lines. **(A)** Chemical structure of VDG. **(B)** Dose-response curves showing the effect of increasing concentrations of VDG on cell viability after 48 h of treatment, assessed by the Alamar Blue HS assay. Data are expressed as the percentage of viable cells relative to untreated controls and presented as mean ± SD (*n* = 3). **(C)** Cell-cycle distribution of cells treated with VDG (12 and 40 μM) for 24 h, analyzed by flow cytometry. Percentages of cells in G1, S, and G2 phases are shown as mean ± SD (*n* = 2). Statistical significance was determined by two-way ANOVA followed by Dunnett's multiple comparisons test, comparing each condition with the untreated control. Symbols indicate: @*p* < 0.05 relative to G1 phase, $*p* < 0.05 relative to S phase, and #*p* < 0.05 relative to G2 phase. **(D)** Representative Western blot analysis of LC3B II, Beclin-1, p62 (SQSTM1), and PARP after 24 h of treatment with VDG (12 and 40 μM). β-Actin was used as a loading control. For p62, an overexposed blot is shown to allow visualization of low-abundance signals where indicated. Densitometric values are shown below each band and represent protein levels normalized to β-actin and expressed as fold changes relative to untreated controls (set to 1). N.D., not detected.

Across four murine tumor cell lines, VDG reduced cell viability in a dose-dependent manner after 48 h, showing broadly comparable response profiles across models (Figure 1B). Comparable analyses performed after 24 h of treatment are shown in Supplementary Figure S1A. Although the tested concentration range resulted in only moderate reductions in viability, VDG induced shifts in cell-cycle distribution in B16 melanoma cells, characterized by a reduced amount of cells in S-phase and increased proportion of cells in G1-phase, consistent with an antiproliferative effect (Figure 1C).

Western blot analysis revealed modest and cell line-dependent changes in autophagy- and apoptosis-related markers following VDG treatment (Figure 1D). LC3B II levels increased significantly in 4T1 and B16 cells, with a more moderate effect in LLC1 cells, whereas Colon-26 cells remained largely unchanged. Beclin-1 levels were modestly increased in B16 and LLC1 cells, remained unchanged in Colon-26 cells, and were reduced in 4T1 cells. p62 was not detected in 4T1 cells under the regular exposure conditions, while in the other cell lines, it remained largely unchanged in B16 and LLC1 cells and was reduced in Colon-26 cells. Total PARP levels decreased in 4T1, B16, and LLC1 cells, whereas they increased in Colon-26 cells. Overall, these findings indicate heterogeneous and moderate activation of autophagy- and apoptosis-related pathways across the tested tumor models, consistent with a generalized cellular stress response rather than robust induction of autophagy or programmed cell death.

**Figure 2:**
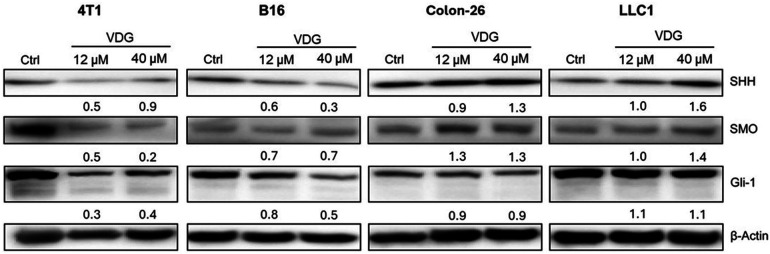
Effects of VDG on hedgehog signaling in 4T1, B16, colon-26, and LLC1 murine cancer cell lines. Representative Western blot analysis of Hedgehog pathway-related proteins after 24 h of treatment with VDG (12 and 40 μM). β-Actin was used as a loading control. Densitometric values are shown below each band and represent protein levels normalized to β-actin and expressed as fold changes relative to untreated controls (set to 1).

Western blot analysis of Hh pathway components revealed cell line-dependent responses to VDG (Figure 2). In B16 cells, treatment reduced SHH and Gli-1 protein levels, while SMO levels were also decreased relative to untreated controls and remained similar between the two tested concentrations. In 4T1 cells, VDG also reduced SMO and Gli-1 levels, whereas SHH showed a less consistent pattern. In contrast, Colon-26 and LLC1 cells did not show a reduction in Gli-1 expression; instead, SHH and/or SMO levels were maintained or increased. Overall, among the four murine tumor models, B16 cells displayed the clearest pattern consistent with Hh pathway inhibition in response to VDG.

Based on the overall response to VDG across the different tumor models, together with the more evident downstream Hh modulation and antiproliferative effects observed in B16 cells, melanoma was selected as a relevant model for subsequent studies. In addition, given the clinical relevance of cutaneous tumors and their accessibility to localized treatment, we next explored the incorporation of VDG into UDL, which have previously been shown to enhance skin penetration and support topical drug delivery.

### 3.2 Enhanced biological effects of UDL-VDG in melanoma cells

**Figure 3:**
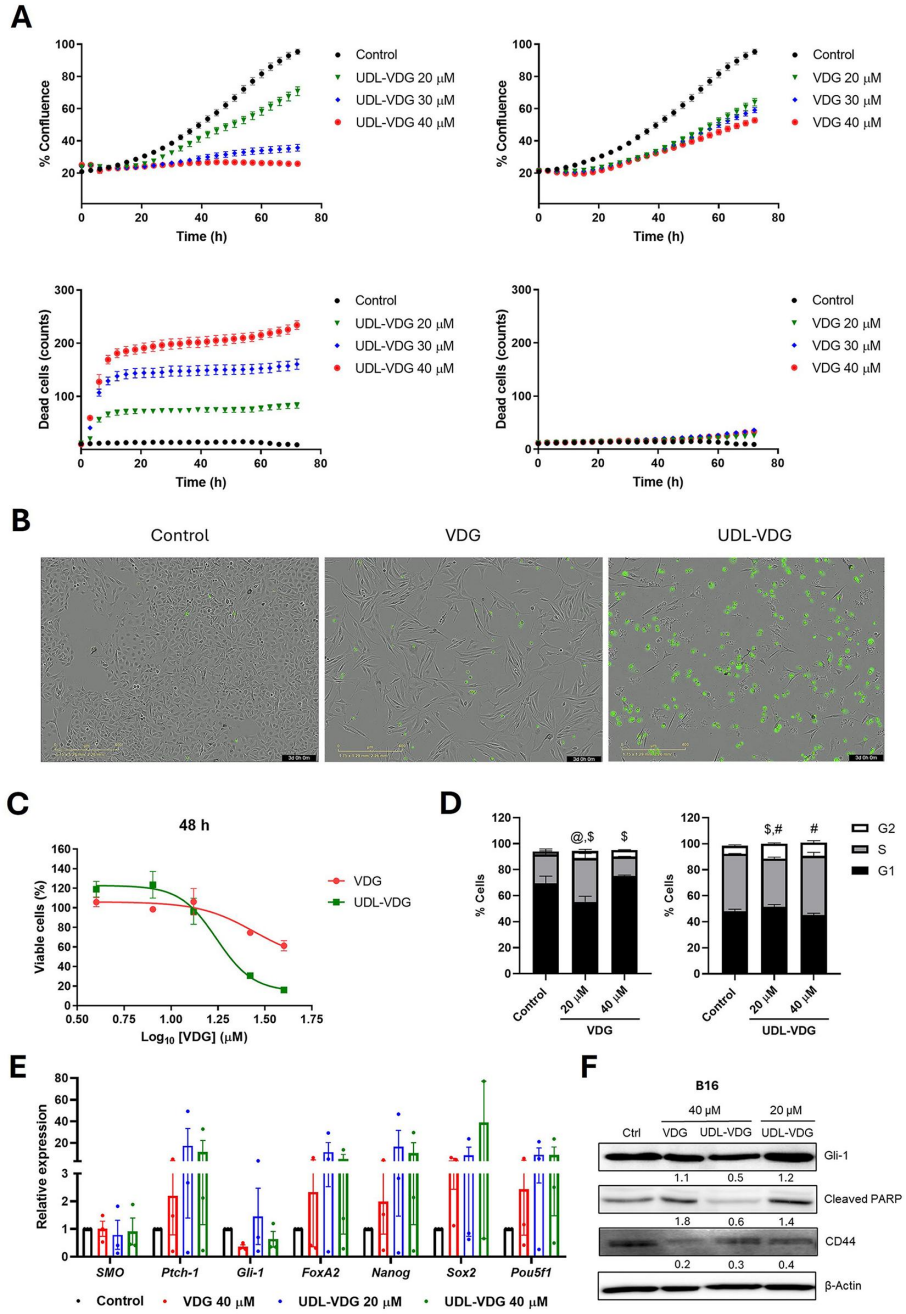
Enhanced antiproliferative and cytotoxic effects of UDL-VDG in B16 melanoma cells and associated modulation of hedgehog signaling and stemness markers. **(A)** Real-time monitoring of cell proliferation (upper panels) and cell death (lower panels; SYTOX Green-positive objects) in cells treated with increasing concentrations of UDL-VDG (left) or free VDG (right). Cells were continuously exposed to the indicated concentrations and monitored for up to 72 h using a live-cell imaging system. Data are presented as mean ± SD (*n* = 3). **(B)** Representative live-cell images acquired at 72 h showing untreated control cells and cells treated with VDG or UDL-VDG (40 μM). Green fluorescence indicates SYTOX Green-positive dead cells. The scale bar corresponds to 400 μm. **(C)** Dose-response curves showing the effect of free VDG and UDL-VDG on cell viability after 48 h of treatment, assessed by the Alamar Blue HS assay. Data are expressed as the percentage of viable cells relative to untreated controls and presented as mean ± SD (*n* = 3). **(D)** Cell-cycle distribution of cells treated with free VDG or UDL-VDG (20 and 40 μM) for 24 h, analyzed by flow cytometry. Percentages of cells in G1, S, and G2 phases are shown as mean ± SD (*n* = 2). Statistical significance was determined by two-way ANOVA followed by Dunnett's multiple comparisons test, comparing each condition with the untreated control. Symbols indicate: @*p* < 0.05 relative to the G1 phase; $*p* < 0.05 relative to the S phase; and #*p* < 0.05 relative to the G2 phase. **(E)** Relative mRNA expression of Hedgehog pathway- and stemness-related genes in cells treated with free VDG or UDL-VDG (20 and 40 μM) for 24 h, as determined by RT-qPCR. Gene expression was calculated using the ΔΔCt method and expressed relative to untreated controls. Data are presented as mean ± SEM (*n* = 3). **(F)** Representative Western blot analysis of Gli-1, cleaved PARP, and CD44 after 24 h of treatment with free VDG or UDL-VDG (20 and 40 μM). β-Actin was used as a loading control. Densitometric values are shown below each band and represent protein levels normalized to β-actin and expressed as fold changes relative to untreated controls (set to 1).

Encapsulation of VDG into UDL altered its biological activity in B16 melanoma cells, resulting in stronger antiproliferative and cytotoxic effects compared with the free drug. Real-time live-cell imaging revealed distinct effects of free VDG and UDL-VDG on cell population dynamics (Figure 3A). UDL-VDG induced a marked and concentration-dependent inhibition of cell growth. At the highest concentration tested (40 μM), UDL-VDG almost completely prevented cell population expansion. In contrast, treatment with free VDG produced only a moderate attenuation of proliferation, with cells continuing to increase in confluence over time. Consistent with these observations, UDL-VDG treatment resulted in a pronounced accumulation of SYTOX Green-positive cells, indicating increased cell death in a dose-dependent manner. Free VDG also induced cell death, but to a lesser extent and with a much slower kinetic profile.

Representative images acquired after 72 h of treatment further supported the differences observed in the real-time analysis (Figure 3B). Untreated B16 cells formed a dense monolayer with minimal SYTOX Green staining. Free VDG (40 μM) moderately reduced cell density and induced the appearance of scattered dead cells. In contrast, UDL-VDG treatment resulted in a marked reduction in cell density and a substantial accumulation of SYTOX Green-positive cells.

Consistent with these observations, cytotoxicity assays confirmed that encapsulation of VDG into UDL increased its potency in B16 cells (Figure 3C). Dose-response curves obtained after 48 h of treatment showed a more pronounced reduction in cell viability for UDL-VDG compared with free VDG, with an IC_50_ value of 17.45 μM for the liposomal formulation. The IC_50_ for free VDG, was not reached within the concentration range tested (4–40 μM). Additional dose-response analyses performed at earlier time points in B16 cells are presented in Supplementary Figure S1B, showing time-dependent differences in the response to VDG and UDL-VDG.

Cell-cycle analysis performed after 24 h of treatment revealed distinct responses to free VDG and UDL-VDG (Figure 3D). Treatment with free VDG at 40 μM significantly reduced the proportion of cells in S phase, suggesting decreased entry into DNA synthesis. In contrast, treatment with UDL-VDG at the same concentration resulted in a significant increase in the fraction of cells in G2 phase relative to untreated controls. These findings indicate that liposomal encapsulation alters the impact of VDG on cell-cycle progression.

The transcriptional effects of free VDG and UDL-VDG were assessed by RT-qPCR analysis of Hh pathway- and stemness-related genes (Figure 3E). Although no statistically significant changes were detected at the analyzed time point, a trend toward reduced Gli-1 expression was observed following treatment with free VDG and UDL-VDG at 40 μM. In contrast, SMO expression remained largely unchanged across treatments, whereas the other analyzed genes, particularly stemness-related markers, showed greater variability.

On a similar note, western blot analysis of selected Hh-, apoptosis-, and stemness-related markers is shown in Figure 3F. In B16 cells, UDL-VDG at 40 μM reduced Gli-1 levels, whereas free VDG at the same concentration did not alter Gli-1 expression. No reduction was observed with UDL-VDG at 20 μM, indicating that this effect was only evident at the higher liposomal concentration tested. Cleaved PARP showed a non-uniform pattern, increasing after free VDG at 40 μM and UDL-VDG at 20 μM, but decreasing after UDL-VDG at 40 μM. CD44 levels were lower than control under all treatment conditions.

**Figure 4:**
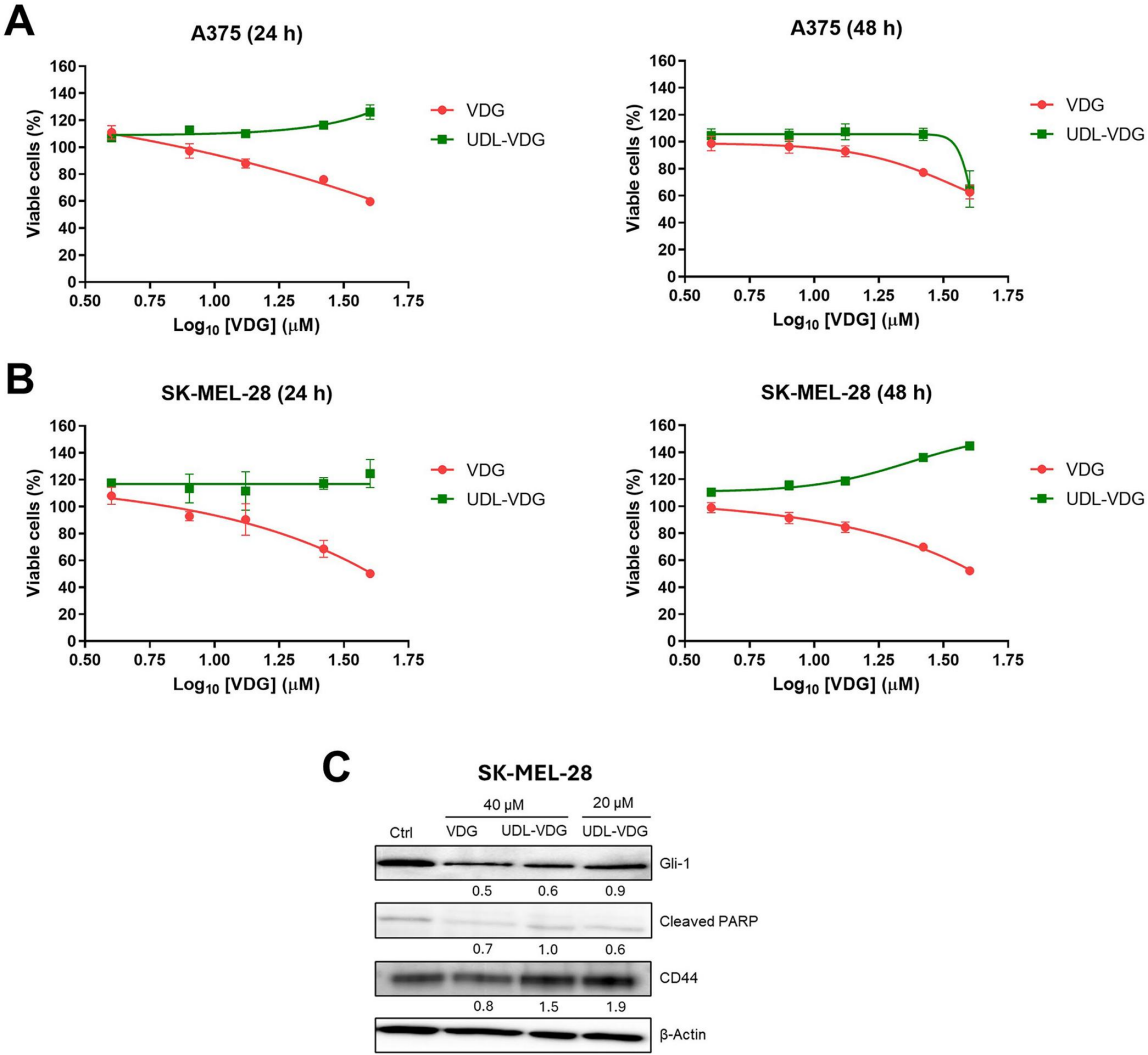
Effects of free VDG and UDL-VDG on cell viability and protein expression in human melanoma cells. Dose-response curves of **(A)** A375 cells and **(B)** SK-MEL-28 cells treated with free VDG or UDL-VDG for 24 and 48 h. Cell viability was assessed by the Alamar Blue HS assay, and data are expressed as the percentage of viable cells relative to untreated controls and presented as mean ± SD (*n* = 3). **(C)** Representative Western blot analysis of Gli-1, cleaved PARP, and CD44 in SK-MEL-28 cells after 24 h of treatment with free VDG or UDL-VDG (20 and 40 μM). β-Actin was used as a loading control. Densitometric values shown below each band represent protein levels normalized to β-actin and expressed as fold change relative to untreated controls.

Interestingly, UDL-VDG had a different effect in human melanoma cell lines A375 and SK-MEL-28, in which it seemed to not affect and even slightly increase cell viability after 24 and 48 h, respectively (Figures 4A,B). However, western blot analysis in the human melanoma cell line SK-MEL-28 (Figure 4C) showed reduced Gli-1 levels after treatment with free VDG and UDL-VDG at 40 μM, whereas UDL-VDG at 20 μM had no evident effect, similar to what was observed in B16 cells (Figure 3F). In contrast, cleaved PARP remained unchanged with UDL-VDG at 40 μM or decreased under the other treatment conditions, while CD44 displayed a divergent pattern, with a slight decrease after free VDG and increased levels after UDL-VDG treatment. Overall, these findings indicate that Gli-1 modulation was treatment- and cell line-dependent, whereas the effects on cleaved PARP and CD44 varied between the two melanoma models.

As an exploratory nanocarrier strategy, VDG was also complexed with DG4.5 and evaluated in B16 melanoma cells. At the tested concentration (7 μM VDG), the DG4.5:VDG complex did not produce detectable changes in cell viability after 24 or 48 h, and cell-cycle distribution remained comparable to untreated controls (Supplementary Figures S1C,D). It should be noted that these experiments were performed using an equivalent VDG concentration for free VDG, UDL-VDG, and DG4.5:VDG, and no significant differences were detected between the two nanoformulations at this concentration. Because the dendrimer formulation was intentionally evaluated at subtoxic DG4.5 levels, the amount of VDG that could be associated with this nanosystem limited the maximum achievable drug concentration. Therefore, while no detectable biological effects were observed for DG4.5:VDG under the tested conditions, this exploratory arm could not be extended to the higher VDG concentrations evaluated for UDL-VDG.

Overall, these results indicate that encapsulation of VDG into UDL enhances its growth-inhibitory and cytotoxic effects in B16 melanoma cells. Compared with the free drug, UDL-VDG produced stronger inhibition of cell population expansion, increased accumulation of dead cells, and distinct effects on cell-cycle progression. Although no marked transcriptional changes were detected at the analyzed time point, protein-level analyses revealed differential modulation of selected markers following liposomal delivery. Taken together, these findings suggest that encapsulation in UDL modifies the cellular response to VDG and enhances its antiproliferative and cytotoxic effects in melanoma cells.

### 3.3 Discussion

The present study explored the cellular responses to VDG across a panel of murine tumor cell lines and evaluated whether encapsulation into UDL could modify its effects in melanoma cells. Although VDG is clinically approved for the treatment of basal cell carcinoma, aberrant Hh signaling has also been implicated in tumor progression and therapy resistance in several other malignancies (40).

We first assessed the responsiveness of a panel of murine cancer cell lines representing melanoma (B16), mammary carcinoma (4T1), colon carcinoma (Colon-26), and Lewis lung carcinoma (LLC1). These tumor types have been reported to involve Hh signaling in tumor initiation, progression, metastasis, stemness regulation, or therapy resistance, although the activation and functional relevance of this pathway can vary substantially between tumor models and may involve both canonical and non-canonical mechanisms (41, 42). Therefore, this panel was used as an initial screening platform to explore whether pharmacological inhibition of Smoothened by VDG could induce detectable cellular responses across different cancer models.

In our screening, VDG produced only moderate reductions in cell viability across all four murine tumor models, with broadly similar dose-response profiles, and was accompanied by limited and variable changes in autophagy- and apoptosis-related proteins. Taken together, these findings suggest that, under the experimental conditions tested, none of the examined cell lines showed a strong dependence on SMO signaling for short-term survival. The overall pattern is likely consistent with a modest treatment-associated stress response, with evidence of growth inhibition in B16 cells rather than robust cell death. The heterogeneous responses observed across the tested cell lines are consistent with previous reports indicating that Hh signaling can display context-dependent activation in different tumor types and experimental models (41, 43). In many solid tumors, Hh pathway output is not uniformly driven by canonical, tumor cell-autonomous SMO activation, but may instead depend on paracrine tumor-stroma interactions or on non-canonical GLI regulation by other oncogenic pathways (44). Under these conditions, direct pharmacological inhibition of SMO is not necessarily expected to produce strong or homogeneous cytotoxic responses across distinct cancer cell lines grown in monoculture. Accordingly, the present screening should be interpreted as an exploratory comparison of direct cellular responses to VDG, rather than as a definitive ranking of Hh pathway dependency across tumor types.

In this context, B16 melanoma cells displayed the clearest biological response, as VDG shifted cell-cycle distribution toward G1 with a concomitant reduction in S phase, consistent with an antiproliferative effect despite the absence of a marked loss of viability. This interpretation is further supported by the analysis of Hh-related proteins, since B16 cells showed the most coherent molecular pattern in response to VDG, with reduced SHH and Gli-1 levels together with lower SMO expression relative to untreated controls. Although these data do not demonstrate a strict dependency on Hh signaling, they indicate that B16 was the most informative model in our panel for detecting both downstream pathway modulation and a measurable antiproliferative response to SMO inhibition. This is in line with previous studies supporting a role of Hh signaling in melanoma biology (45). Moreover, the cutaneous localization of non-metastatic melanoma lesions makes this tumor type particularly suitable for exploring localized drug delivery strategies.

UDL, also known as transfersomes, represent a specialized class of elastic nanovesicles capable of enhancing drug penetration across the *stratum corneum* and have been widely investigated as carriers for topical drug delivery (46, 47). Previous work from our group has also demonstrated that UDL-VDG improves drug penetration in *ex vivo* human skin models (31). Therefore, we investigated whether the incorporation of VDG into UDL could influence its cellular effects in melanoma cells.

The incorporation of VDG into UDL clearly modified its biological effects in B16 melanoma cells. Compared with the free drug, UDL-VDG produced a stronger, concentration-dependent inhibition of cell population expansion and accumulation of dead cells, together with a distinct effect on cell-cycle distribution, with enrichment in G2 rather than the reduction in S phase observed with free VDG. These findings indicate that liposomal delivery did not simply increase the magnitude of the response to VDG, but altered its phenotypic profile. In this regard, UDL-VDG was associated with a more pronounced cytotoxic response under the conditions tested, and a much more pronounced cytostatic effect compared to free VDG.

At the mechanistic level, however, the relationship between these phenotypic effects and Hh pathway inhibition was less straightforward. While no statistically significant transcriptional changes were detected at the analyzed time point, the RT-qPCR and the protein data suggested some degree of pathway modulation, particularly at the higher drug concentration. In B16 cells, UDL-VDG at 40 μM reduced Gli-1 protein levels, whereas free VDG at the same concentration did not. In SK-MEL-28 cells, both free VDG and UDL-VDG at 40 μM decreased Gli-1. Taken together, these observations support the idea that VDG-based treatments were able to modulate Hh output in melanoma cells, but they do not indicate that the stronger cytotoxic effect of UDL-VDG can be fully explained by canonical Hh inhibition alone.

An additional, and likely complementary, explanation is that liposomal encapsulation may alter the intracellular pharmacology of VDG independently of the extent of canonical Hh inhibition. Liposomal carriers can modify the way drugs interact with the plasma membrane, increase cellular association and uptake, and alter intracellular trafficking through endosomal and lysosomal compartments, thereby altering drug availability in relevant subcellular compartments (48). The cellular internalization of nanocarriers is influenced by both nanocarrier physicochemical properties—such as size, shape, surface charge, surface chemistry, hydrophobicity, and elasticity—and by the properties of the recipient cell membrane, which together determine uptake efficiency and the intracellular entry pathways preferentially engaged (49, 50). In melanoma cells, liposomal uptake has been reported to be formulation-dependent and may involve clathrin-mediated endocytosis, caveolae-mediated endocytosis, and/or macropinocytosis, indicating that carrier composition can substantially influence intracellular delivery routes (51). In addition, our previous work in SK-MEL-28 cells showed that, although UDL-VDG uptake was more pronounced at 37 °C, a non-negligible signal was still detected at 4 °C, suggesting that, besides predominantly endocytic uptake, direct nanocarrier-cell interactions or other energy-independent contributions cannot be entirely excluded (27). In this context, the stronger cytotoxic phenotype observed with UDL-VDG may reflect not only partial modulation of Hh output, but also carrier-dependent differences in membrane interaction, internalization, vesicular trafficking, and intracellular drug release. This possibility is particularly relevant for Hh-directed therapies, since SMO activity is known to depend on the membrane lipid environment and sterol availability, including pools of accessible cholesterol that contribute to pathway regulation (52, 53). Therefore, changes in membrane interactions and intracellular trafficking induced by UDL could, in principle, influence both the effective intracellular exposure to VDG and the lipid environment in which Hh signaling is regulated. Although these mechanisms were not directly addressed in the present study, they provide a plausible framework to explain why UDL-VDG generated a stronger cytotoxic response than the free drug despite only partial evidence of pathway modulation at the transcriptional and protein levels.

Furthermore, although no other specific targets of VDG have been thoroughly identified, we cannot rule out the possibility that part of its biological activity may involve off-target interactions with other modulators of cell viability. On that line, COX2 has recently been proposed as a potential target candidate for VDG binding through deep-learning-based drug repositioning and molecular docking analyses, warranting further research (54).

Interestingly, different responses to UDL-VDG were observed across several melanoma models, in the present study, with B16 cells showing the most sensitivity to UDL-VDG, whereas A375 and SK-MEL-28 cells did not reproduce the same response pattern in the Alamar Blue assay, further supporting a cell line-dependent effect. Our previous work in SK-MEL-28 cells showed that UDL-VDG produced greater cytotoxicity than the free drug at early time points and increased apoptosis induction under the tested conditions (27). Although those experiments were performed using different viability assays and readouts than the present study, they support the general notion that liposomal delivery can enhance the biological activity of VDG in at least some melanoma settings. At the same time, the more limited response observed here in SK-MEL-28 and the distinct behavior of A375 further indicate that the magnitude and profile of the response are strongly model- and assay-dependent. This heterogeneity is consistent with previous melanoma studies indicating that Hh-GLI signaling contributes to melanoma biology only in a subset of models and that sensitivity to SMO inhibition is variable rather than universal (55). Moreover, such variability may also reflect differences in nanocarrier-cell interactions, as previous studies have shown that the same lipid-based delivery system can engage distinct uptake pathways in different melanoma cell lines, which may in turn influence the intracellular handling of the encapsulated drug. In this regard, Pautu et al. reported differential internalization mechanisms in B16F10 and SK-MEL-28 melanoma cells exposed to the same lipid nanocarrier system, supporting the notion that intracellular trafficking of lipid-based nanocarriers is highly cell line-dependent (56).

On the other hand, an exploratory evaluation of DG4.5 as an alternative carrier did not reveal detectable effects on B16 viability or cell-cycle progression at the tested concentration. DG4.5 was selected based on our previous findings showing that it can penetrate the *stratum corneum* and transport VDG to deeper layers of the skin, while improving its solubility in aqueous media (32). Importantly, viability and cell-cycle analyses in this exploratory arm were performed at an equivalent VDG concentration for UDL-VDG and DG4.5:VDG, and no significant differences were detected between the two nanoformulations at this concentration. Since the formulation was intentionally evaluated at subtoxic DG4.5 levels, the lack of detectable biological effects is likely related to the limited VDG concentration that could be achieved in the cell assays.

Overall, these findings support the potential of UDL-VDG as a topical delivery strategy for candidate non-metastatic cutaneous melanoma lesions, in line with our previous studies showing improved skin penetration of this formulation (31), and indicate that liposomal encapsulation can potentiate and modulate the *in vitro* anti-melanoma activity of VDG. At the same time, the present results indicate that the underlying mechanism remains only partially resolved. The available data are compatible with some contribution of Hh pathway modulation, particularly through Gli-1 reduction at the higher concentration, yet they do not conclusively allow attributing the enhanced cytotoxic effect of UDL-VDG solely to canonical Hh inhibition. Additional experiments are warranted to define more precisely how liposomal delivery reshapes the pharmacological response to VDG in melanoma cells.

## 4 Conclusions

In conclusion, this study shows that VDG elicits measurable but overall moderate cellular responses across different murine tumor models, highlighting the context-dependent nature of its activity outside basal cell carcinoma. Among the cell lines evaluated, B16 melanoma emerged as the most informative model, showing evidence of growth inhibition together with modulation of Hh-related proteins in response to free VDG.

Importantly, encapsulation of VDG into UDL markedly altered its biological effects in melanoma cells. Compared with the free drug, UDL-VDG produced stronger inhibition of cell population expansion and increased accumulation of dead cells, supporting the notion that liposomal delivery potentiates the *in vitro* anti-melanoma activity of VDG. At the same time, the molecular data indicate that this enhanced effect cannot be attributed solely to canonical Hh inhibition, although modulation of Gli-1 at higher concentrations suggests that Hh pathway regulation may contribute, at least in part, to the observed response.

Taken together, these findings support UDL as a promising strategy to improve the delivery and biological activity of VDG in candidate non-metastatic cutaneous melanoma settings. They also suggest that the incorporation of VDG into nanocarrier systems may broaden the possibilities for its repurposing beyond its current clinical use and could potentially contribute to improving efficacy and optimizing the safety profile. However, the variability observed across melanoma models and the incomplete mechanistic resolution indicate that further studies will be needed, particularly *in vivo* and in a broader panel of tumor cell lines, to clarify how these formulations reshape VDG activity and to better define their repurposing potential.

## Data Availability

The raw data supporting the conclusions of this article will be made available by the authors, without undue reservation.
