# Supplementary material for: Ultradeformable liposomal delivery of vismodegib modulates its biological activity in melanoma cells
Source: Front Med Technol. 2026 Jun 5;8:1826970. doi: 10.3389/fmedt.2026.1826970 (PMC13295005; doi:10.3389/fmedt.2026.1826970)
Supplement: Supplementary file 1 [file Supplementaryfile1.docx]

***Supplementary Material***

# Detailed protocol for the preparation and characterization of DG4.5:VDG complexes

DG4.5:VDG complexes were prepared by combining one volume of DG4.5 (0.012 mM) with one volume of VDG (1.2 mM), both dissolved in methanol. The mixture was stirred for 24 h at 100 rpm at room temperature to allow complex formation. Subsequently, the solvent was removed using a Savant AES1010 SpeedVac concentrator (Thermo Fisher Scientific, MA, USA). The sample was then rehydrated with 10 mM phosphate-buffered saline (PBS) to obtain an aqueous suspension. To remove non-complexed drug, the sample was centrifuged at 10,000 rpm, and the supernatant was collected.

Formation of the complexes was confirmed by physicochemical characterization, including quantification of VDG in the complexes, particle size, and zeta potential.

# Supplementary Figure and Tables

## Supplementary Tables

**Supplementary Table 1. Antibodies used for Western blot experiments.**

|  | **Target Protein** | **Host** | **Source** | **Identifier** |
| --- | --- | --- | --- | --- |
| **Primary antibodies** | Beclin-1 | Rabbit | ABclonal | A7353 |
|  | Cleaved PARP | Rabbit | Cell Signaling Technology | 5625 |
|  | GLI-1 (C-1) | Mouse | Santa Cruz Biotechnology | sc-515751 |
|  | LC3B | Rabbit | Cell Signaling Technology | 3868S |
|  | p62 (SQSTM1) | Rabbit | Santa Cruz Biotechnology | sc-25575 |
|  | PARP | Rabbit | Cell Signaling Technology | 9532 |
|  | Shh (E-1) | Mouse | Santa Cruz Biotechnology | sc-365112 |
|  | Smo (E-5) | Mouse | Santa Cruz Biotechnology | sc-166685 |
|  | β-Actin-HRP | Mouse | Santa Cruz Biotechnology | sc-47778 |
| **Secondary antibodies** | Goat anti-Mouse IgG (H+L), HRP-conjugated | Goat | Thermo Fisher Scientific | 31430 |
|  | Goat anti-Rabbit IgG (H+L), HRP-conjugated | Goat | Thermo Fisher Scientific | 31460 |

**Supplementary Table 2. Primer sequences for RT-qPCR.**

| **Gene** | **Forward sequence** | **Reverse sequence** |
| --- | --- | --- |
| Gli-1 | CTCAAACTGCCCAGCTTAACCC | TGCGGCTGACTGTGTAAGCAGA |
| Smo | GAGGCTACTTCCTCATCAGAGG | GCTGAAGGTGATGAGCACAAAGC |
| Ptch1 | CCTCGCTTACAAACTCCTGGTG | TGATGCCATCTGCGTCTACCAG |
| FoxA2 | CGAGCACCATTACGCCTTCAAC | AGTGCATGACCTGTTCGTAGGC |
| Nanog | GAACGCCTCATCAATGCCTGCA | GAATCAGGGCTGCCTTGAAGAG |
| Sox2 | AACGGCAGCTACAGCATGATGC | CGAGCTGGTCATGGAGTTGTAC |
| Pou5f1 | CAGCAGATCACTCACATCGCCA | GCCTCATACTCTTCTCGTTGGG |

**Supplementary Table 3. Physicochemical characteristics of UDL-VDG and DG4.5:VDG formulations.**

| **Formulation** | **VDG (mM)** | **Size (nm)*** | **Polydispersity Index (PDI)** | **Zeta potential (mV)** |
| --- | --- | --- | --- | --- |
| **UDL** | - | 108.0 ± 2.0 | 0.04 ± 0.02 | -19.75 ± 1.02 |
| **UDL-VDG** | 3.06 ± 0.23 | 114.2 ± 1.3 | 0.05 ± 0.01 | -17.80 ± 0.85 |
| **DG4.5** | - | 498.0 ± 185.2 | 0.42 ± 0.11 | -38.21 ± 2.04 |
| **DG4.5:VDG** | 0.060 ± 0.002 | 170.0 ± 1.3 | 0.28 ± 0.01 | -36.21 ± 1.84 |

*Size corresponds to intensity-weighted hydrodynamic diameter determined by Dynamic Light Scattering.

Values are expressed as mean ± SD (n = 3).

## Supplementary Figure


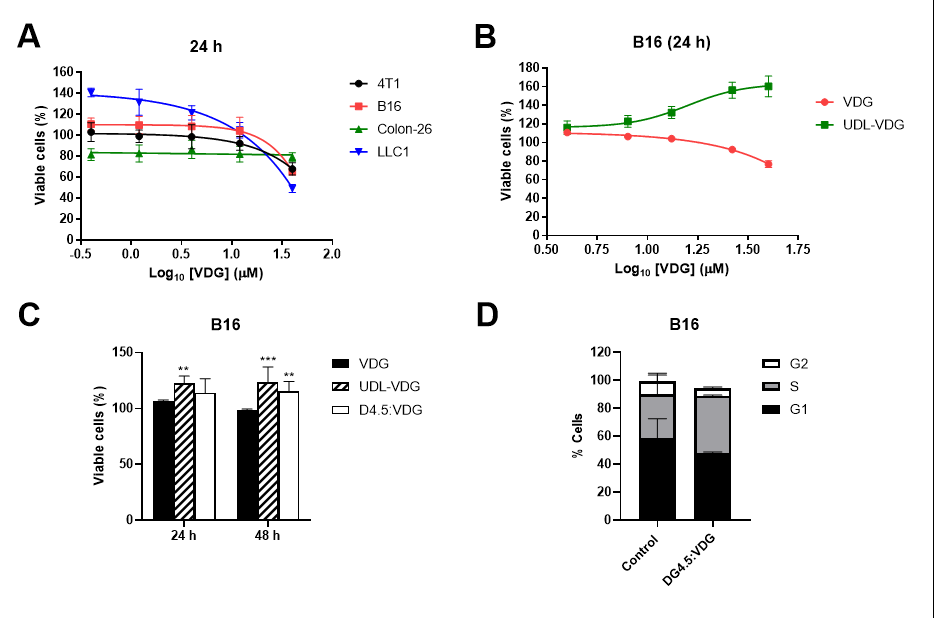


**Supplementary Figure 1. Effects of free and VDG-loaded UDL or DG4.5 on cell viability and cell-cycle distribution in murine tumor cells.** **(A)** Dose-response curves showing the effect of increasing concentrations of VDG on cell viability in 4T1, B16, Colon-26, and LLC1 cell lines after 24 h of treatment. **(B)** Dose-response curves of B16 cells treated with free VDG or UDL-VDG for 24 h. In **(A)** and **(B)**, cell viability was assessed by the Alamar Blue HS assay, and data are expressed as the percentage of viable cells relative to untreated controls and are presented as mean ± SD (n = 3). **(C)** Cell viability of B16 cells after 24 h and 48 h of incubation with free VDG, UDL-VDG, or DG4.5:VDG, assessed by the Alamar Blue HS assay. Treatments were performed using equivalent VDG concentrations (7 μM), corresponding to 1.2 μM DG4.5 dendrimer in the conjugated formulation. Data are expressed as the percentage of viable cells relative to untreated controls and are presented as mean ± SD (n = 2). Statistical significance was determined by two-way ANOVA followed by Tukey’s multiple comparisons test. All pairwise comparisons were performed; however, statistically significant differences were observed only when nanoformulations were compared with the free drug, whereas no significant differences were detected between UDL-VDG and DG4.5:VDG. ***p* < 0.01, ****p* < 0.001. **(D)** Cell-cycle distribution of B16 cells treated with DG4.5:VDG at a VDG concentration of 7 μM, analyzed by flow cytometry after 24 h. The percentages of cells in G1, S, and G2 phases are shown as mean ± SD (n = 2). Statistical significance was determined by two-way ANOVA followed by Sidak’s multiple comparisons test. No significant differences in cell-cycle distribution were observed relative to untreated controls.
